# Efficacy of PRaG therapy in microsatellite-stable metastatic colorectal cancer: a comparative analysis of PD-1/PD-L1 inhibitor-based combination therapies

**DOI:** 10.1007/s00262-025-04185-y

**Published:** 2025-10-06

**Authors:** Jiabao Yang, Xiangrong Zhao, Pengfei Xing, Yuehong Kong, Meiling Xu, Liyuan Zhang

**Affiliations:** 1 Center of PRaG Therapy, The Second Affiliated Hospital of Soochow University, Suzhou, China; 2 Department of Radiotherapy, The First Affiliated Hospital of Xi′an Jiaotong University, Xi′an, China

**Keywords:** MSS/pMMR, Colorectal cancer, PD-1/PD-L1 inhibitor, Radiotherapy, Retrospective study

## Abstract

**Background:**

Most (95%) metastatic colorectal cancers (mCRCs) exhibit microsatellite stability (MSS) and proficiency in DNA mismatch repair (pMMR), leading to resistance to immunotherapy. To overcome this, the combination of programmed cell death protein 1 (PD-1)/programmed cell death ligand 1 (PD-L1) inhibitors with other therapeutic strategies has been explored. This study evaluated the efficacy of PRaG therapy—a combination of PD-1 inhibitors, radiotherapy, and granulocyte‒macrophage colony-stimulating factor—in comparison with other combination therapies.

**Methods:**

MSS/pMMR mCRC patients receiving PD-1/PD-L1 inhibitor combination therapy at the Second Affiliated Hospital of Soochow University were analyzed. Patients were grouped by therapy type, and SPSS 26.0 was used to assess clinical features, survival outcomes, tumor response, and adverse reactions.

**Results:**

A total of 101 patients were enrolled and categorized into three groups: a PD-1/PD-L1 inhibitor combined with chemotherapy group (n = 35), a tyrosine kinase inhibitor group (n = 36), and a PRaG therapy group (n = 30). Clinical outcomes, including median progression-free survival (mPFS), median overall survival (mOS), response rates, and prognostic factors, were analyzed using SPSS 26.0. The prognostic analyses being conducted only in the PRaG therapy group. PRaG therapy group had the highest objective response rate (20.0%) and the longest mPFS (4.5 months, *P* < 0.05). However, no significant difference in mOS was observed among the groups. Prognostic analysis identified baseline peripheral lymphocyte counts, CD8 + T cell counts, and IL-17A levels as independent factors influencing treatment outcomes.

**Conclusions:**

PD-1/PD-L1 inhibitor-based therapies show clinical efficacy in MSS/pMMR mCRC patients. PRaG therapy has potential advantages over chemotherapy or tyrosine kinase inhibitors, with CD8 + T cell counts and IL-17A levels being key prognostic markers.

**Supplementary Information:**

The online version contains supplementary material available at 10.1007/s00262-025-04185-y.

## Introduction

The prognosis of MSS mCRC is generally poor following the failure of second-line treatments [1]. Approximately 95% of mCRCs are MSS/pMMR, thereby affecting a large group of patients [2]. Regorafenib and trifluridine-tipiracil (TAS-102) are commonly recommended for postline treatment of MSS/pMMR mCRC; however, the survival benefit remains limited [3]. Increasing treatment efficacy for patients with advanced MSS/pMMR mCRC is a critical focus of ongoing clinical research. One potential strategy is the use of combined therapies, which may convert “cold tumors,” which are resistant to immunotherapy, into “hot tumors,” thereby increasing their susceptibility to immune-based treatments [4]. Previous studies have demonstrated that chemotherapy, molecular targeted therapy, and radiotherapy can increase the effectiveness of immunotherapy for MSS/pMMR mCRC [5–7].

In accordance with tumor immune response principles, our research group independently designed and registered a clinical trial (registration number: ChiCTR1900026175) to investigate the efficacy of triple-modality therapy involving PD-1 inhibitors, radiotherapy, and GM-CSF in patients with advanced solid tumors. The treatment regimen, derived from the combination of a “PD-1 inhibitor, Radiotherapy, and GM-CSF,” is termed PRaG therapy [8]. Preliminary results indicate that PRaG therapy has therapeutic effects for a subset of patients, including those who have previously failed single-agent PD-1 blockade therapy. Further analysis revealed that the PRaG regimen may improve clinical outcomes in MSS/pMMR mCRC patients, with an acceptable toxicity profile [9, 10]. Compared with other combined radiotherapy and immunotherapy regimens evaluated in clinical trials, PRaG therapy has demonstrated a significant advantage in terms of survival outcomes [11]. To date, the PRaG treatment regimen has been widely adopted by multiple hospitals, with numerous successful treatment cases reported [12–16].

Patients with MSS/pMMR metastatic colorectal cancer (mCRC) typically have limited sensitivity to immunotherapy [2]. In the KEYNOTE-016 and KEYNOTE-028 studies, the ORR for MSS/pMMR mCRC patients treated with pembrolizumab was 0% [17, 18]. Recently, efforts to combine PD-1/PD-L1 inhibitors with other therapeutic modalities, such as chemotherapy, targeted therapy, or radiotherapy, have been explored to overcome the resistance of MSS/pMMR mCRC to immunotherapy. Both the BACCI and REGONIVO studies have shown that immunotherapy combined with chemotherapy or targeted therapy provides only limited efficacy in MSS/pMMR mCRC patients [7, 19, 20]. There is still a paucity of prospective clinical trials evaluating the combination of immunotherapy and radiotherapy, and the efficacy of this approach for the treatment of MSS/pMMR mCRC remains unclear [6]. Furthermore, there are currently no clinical guideline-recommended therapies for the postline treatment of MSS/pMMR mCRC. In our institution’s prospective clinical trial of a triple-combination therapy, we found that PRaG treatment may offer therapeutic benefits for patients with colorectal cancer. In this study, we further investigated the role of PRaG therapy in MSS/pMMR mCRC patients and analyzed its potential impact on survival outcomes. Additionally, patients with advanced MSS/pMMR mCRC typically have a poor outcome, characterized by a short expected survival time, poor general health, and limited treatment tolerance. According to previous clinical data, chemotherapy often results in significant adverse reactions, whereas targeted therapies show limited efficacy. In contrast, PRaG therapy is associated with a relatively low incidence of adverse events and demonstrates certain efficacy, making it a promising avenue for further exploration.

This study aims to conduct a retrospective analysis of the efficacy and safety of PRaG therapy in patients with MSS/pMMR metastatic colorectal cancer, and to explore the related prognostic factors. At the same time, the therapeutic effect of PRaG therapy will be compared with other commonly used combined treatment protocols.

## Materials and methods

This retrospective study has been approved by the Ethics Committee of the Second Affiliated Hospital of Soochow University. Patients with advanced MSS/pMMR mCRC treated with combined PD-1/PD-L1 inhibitor therapy at the Second Affiliated Hospital of Soochow University between January 2018 and September 2022 were eligible for enrollment. The specific combination therapy regimens were formulated by the attending physicians based on the patients’ individual conditions and clinical characteristics.

We collected the clinical data of eligible patients in our hospital and divided them into three groups based on the different treatment regimens they received: PD-1/PD-L1 inhibitor combined with chemotherapy, combined molecular targeted therapy and combined radiotherapy. The patients with PD-1/PD-L1 inhibitor combined with fluorouracil-based chemotherapy were selected as the combined chemotherapy group, and the patients treated with PD-1/PD-L1 inhibitor combined with antiangiogenic tyrosine kinase inhibitor were selected as the combined TKI group. The patients who received radiotherapy combined with PD-1/PD-L1 inhibitor and GM-CSF with or not IL-2 (PRaG regimen) were taken as the combined radiotherapy group (PRaG therapy group). All cancers were confirmed as MSS/pMMR by immunohistochemistry or next-generation sequencing (NGS). Distant metastasis was confirmed by biopsy or imaging studies.

The clinical data collected included patient demographics (age and sex), Eastern Cooperative Oncology Group (ECOG) Performance Status score, routine blood test results before and during treatment, primary cancer sites, and the presence or absence of celiac effusion. Absolute counts of peripheral blood lymphocyte subsets and cytokine levels were measured by flow cytometry and recorded. Imaging data (CT, MRI, PET-CT, and ECT) during treatment were collected to evaluate the therapeutic response before and during treatment.

Tumor response was assessed using the Response Evaluation Criteria in Solid Tumors (RECIST 1.1), with outcomes categorized into four types: complete remission (CR), partial response (PR), stable disease (SD), and progressive disease (PD). Adverse reactions were evaluated according to the Common Terminology Criteria for Adverse Events (CTCAE 5.0). The primary outcome measures were progression-free survival (PFS), overall survival (OS), the objective response rate (ORR), the disease control rate (DCR), and adverse events during treatment.

Data analysis was performed using SPSS 26.0 software. X-tile software was used to determine the optimal cutoff values for the baseline peripheral blood lymphocyte count, lymphocyte subset distribution, and cytokine levels with respect to patient outcomes. Chi-square tests or Fisher’s exact tests were used to analyze the impact of clinical features on treatment efficacy. Survival curves were generated using the Kaplan‒Meier method, and differences in survival between groups were compared using the log-rank test. A Cox proportional hazards model was used to evaluate baseline characteristics and their associations with PFS and OS through univariate and multivariate regression analyses to identify independent risk factors for survival. Statistical significance was defined as *P* < 0.05.

## Results

A total of 101 patients were enrolled in this study. The patients have been followed up until December 31, 2022, with a median follow-up time of 12.5 months. Among these patients, 35 patients received a PD-1/PD-L1 inhibitor combined with fluorouracil-based chemotherapy, 36 patients received a PD-1/PD-L1 inhibitor combined with a tyrosine kinase inhibitor, and 30 patients received PRaG therapy. The median age of the patients was 67 years. All patients had moderately to poorly differentiated adenocarcinomas, and all cancers were classified as stage IV (AJCC 8th edition). Prior to initiating combined treatment, all patients received at least two lines of systemic therapy. More than 36% of patients had more than five metastatic lesions. The detailed baseline characteristics are presented in Table 1.

**Table 1:** Baseline patient demographic and clinical characteristics (n = 101)

Characteristic	Overall (n = 101)	Combined with chemotherapy (n = 35)	Combined with TKI (n = 36)	PRaG therapy (n = 30)	*P* value
Age (years)					0.016
Median, range	67 (31, 86)	68 (46, 86)	62 (31, 82)	69.5 (36, 78)	
< 65	43 (42.6%)	10 (28.6%)	22 (61.1%)	11 (36.7%)	
≥ 65	58 (57.4%)	25 (71.4%)	14 (38.9%)	19 (63.3%)	
Sex					0.695
Male	68 (67.3%)	23 (65.7%)	23 (63.9%)	22 (73.3%)	
Female	33 (32.7%)	12 (34.3%)	13 (36.1%)	8 (26.7%)	
Primary cancer sites					0.301
rectum	45 (44.6%)	19 (54.3%)	13 (36.1%)	13 (43.3%)	
colon	56 (55.4%)	16 (45.7%)	23 (63.9%)	17 (56.7%)	
ECOG performance status					0.618
1	34 (33.7%)	9 (25.7%)	14 (38.9%)	11 (36.7%)	
2	46 (45.5%)	17 (48.6%)	17 (47.2%)	12 (40.0%)	
3	21 (20.8%)	9 (25.7%)	5 (13.9%)	7 (23.3%)	
Liver metastasis					0.044
Yes	55 (54.5%)	14 (40.0%)	25 (69.4%)	16 (53.3%)	
No	46 (45.5%)	21 (60.0%)	11 (30.6%)	14 (46.7%)	
Malignant ascites					0.466
Yes	23 (22.8%)	6 (17.1%)	8 (22.2%)	9 (30.0%)	
No	78 (77.2%)	29 (82.9%)	28 (77.8%)	21 (70.0%)	
No. of prior systemic therapies					0.618
2	46(45.6%)	19 (54.3%)	14 (38.9%)	13 (43.3%)	
≥ 3	52(51.5%)	14 (40.0%)	21 (58.3%)	17 (56.7%)	
No. of metastatic site					0.345
2 ~ 5	58 (57.4%)	21 (60.0%)	23 (63.9%)	14 (46.7%)	
> 5	43 (42.6%)	14 (40.0%)	13 (36.1%)	16 (53.3%)	

For continuous variables, normality test was carried out. The data in accordance with normal distribution (age was described by meanÂ ± SD. The distribution of variables that did not conform to the normal distribution (the number of metastatic site and prior systemic therapies) was described by median (P25, P75). The classification variables (sex, ECOG score, Primary cancer sites, liver metastasis, Malignant ascites) were expressed by n (%). For normally distributed data (verified by Shapiro–Wilk tests), we performed one-way ANOVA followed by Tukey's post hoc tests for inter-group comparisons. For non-normally distributed data, Kruskal–Wallis tests with Dunn's post hoc corrections were applied. The p-values in the table represent the omnibus tests (ANOVA/Kruskal–Wallis) for overall group differences

### Evaluation of tumor response and survival in patients

**Fig. 1:**
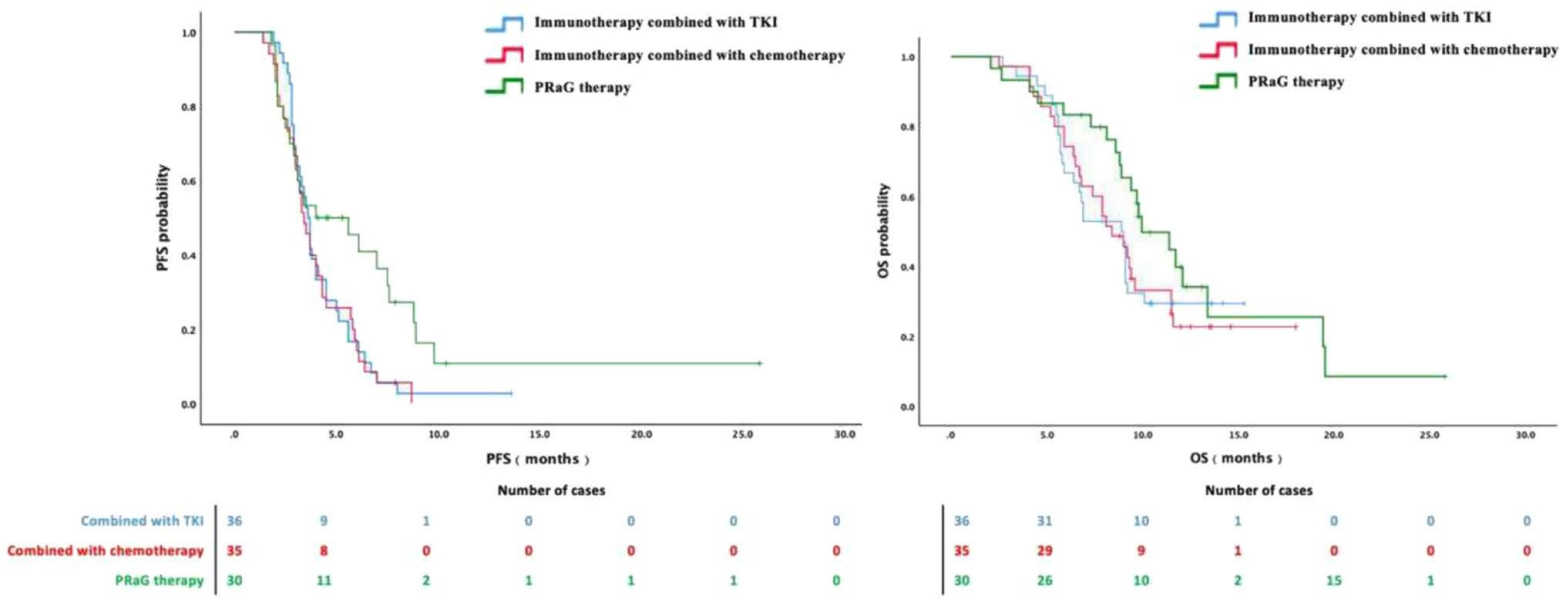
Kaplan–Meier curves of progression-free survival and overall survival

The median mPFS and mOS in the PD-1/PD-L1 inhibitor plus chemotherapy group were 3.1 months (95% CI 2.5–3.7) and 8.4 months (95% CI 6.7–10.1), respectively. In the PD-1/PD-L1 inhibitor plus tyrosine kinase inhibitor group, the mPFS and mOS were 3.4 months (95% CI 3.2–3.6) and 8.9 months (95% CI 7.3–10.5), respectively. The PRaG therapy group presented the longest mPFS (4.5 months, 95% CI 1.2–7.8) and mOS (10.0 months, 95% CI 7.6–12.4). The survival curve is presented in Fig. 1. The mPFS in the PRaG therapy group was significantly longer than that in the other two groups, as determined by the log-rank test (*P* < 0.05). No significant difference in mPFS was observed between the PD-1/PD-L1 inhibitor plus chemotherapy group and the PD-1/PD-L1 inhibitor plus tyrosine kinase inhibitor group. Similarly, no significant differences in mOS were observed among the three groups.

### Evaluation of the curative effect in patients in the three groups

**Fig. 2:**
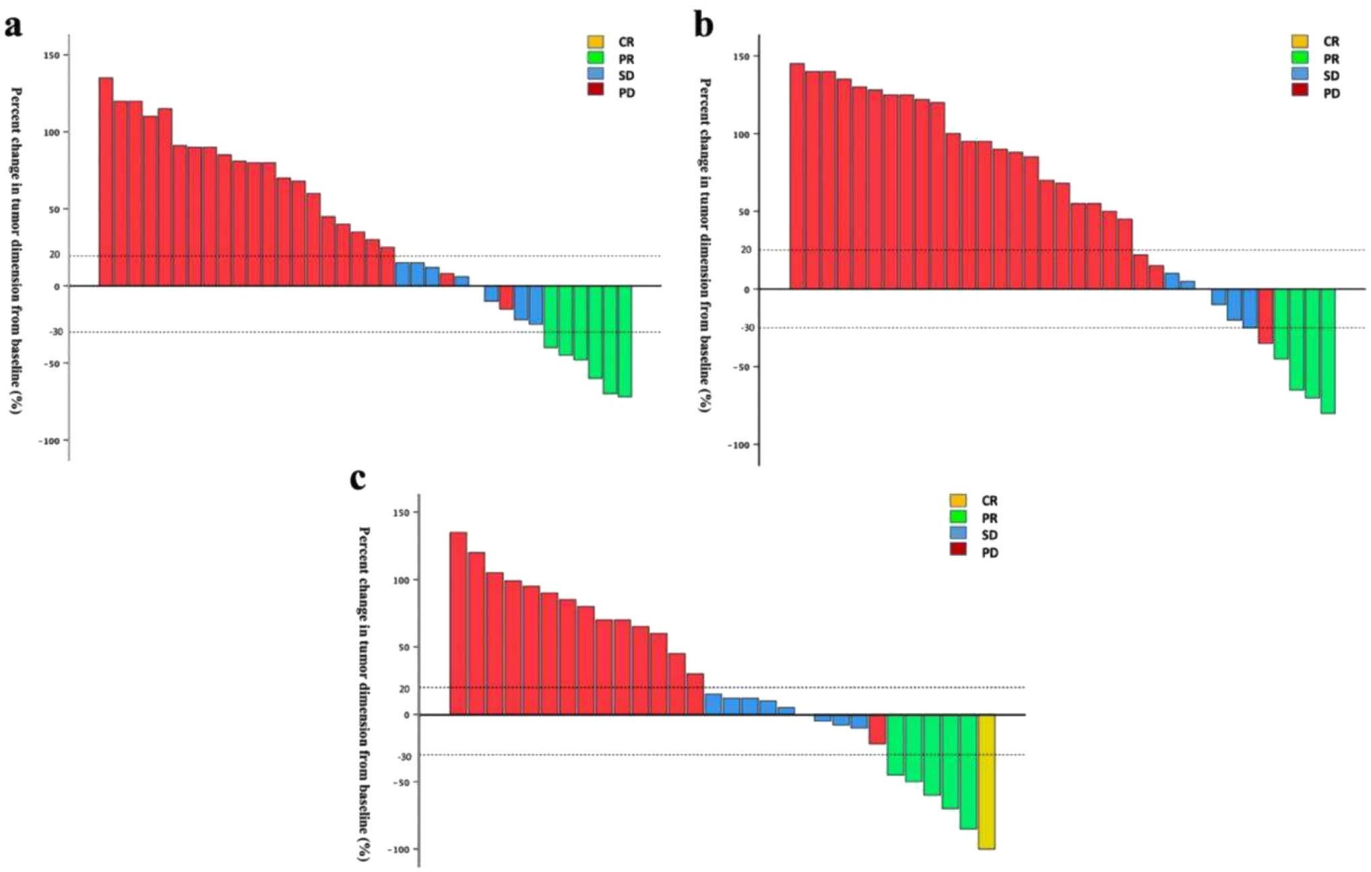
Percent change in tumor dimension of comparable lesion(s) at best responses. **a** Immunotherapy combined with chemotherapy; **b** Immunotherapy combined with TKI; **c** PRaG therapy. Complete remission (CR), Partial response (PR), stable disease (SD), and progressive disease (PD)

The therapeutic response was assessed according to the RECIST 1.1 criteria. The ORRs in the PD-1/PD-L1 inhibitor plus chemotherapy group, PD-1/PD-L1 inhibitor plus tyrosine kinase inhibitor group, and PRaG therapy group were 11.4%, 16.7%, and 20.0%, respectively, while the DCRs were 28.6%, 38.9%, and 50.0%, respectively. The ORR and DCR in the PRaG therapy group were greater than those in the other two groups. See Supplementary Table 1 for details. However, no significant differences were observed among the three groups, likely due to the limited sample size. Figure 2 illustrates the changes in target lesions across the three groups. Notably, only one patient achieved a complete remission, which was observed in the PRaG therapy group.

### Analysis of the PRaG therapy group

All patients in the PD-1/PD-L1 inhibitor combined with radiotherapy group were enrolled in the PRaG clinical trials (ChiCTR1900026175 and NCT04892498). In accordance with the study protocol, the absolute counts of peripheral blood lymphocyte subsets and cytokine levels were measured prior to each treatment cycle. Univariate and multivariate analyses of PFS and OS were conducted on the basis of these measurements. X-tile software was used to determine the critical thresholds for lymphocyte subset counts and cytokine levels, categorizing these variables into high and low groups on the basis of the cutoff values.

Univariate analysis revealed that lower baseline counts of CD8 + T lymphocytes and NK cells were associated with poorer PFS (*P* = 0.007; *P* = 0.044), and higher baseline levels of IL-17A were associated with an increased risk of disease progression (*P* = 0.010). These variables, along with baseline CD8 + T lymphocyte counts, NK cell counts, and IL-17A levels, were included in the multivariate analysis. The results of the multivariate analysis indicated that the baseline CD8 + T lymphocyte count was an independent prognostic factor for PFS in patients treated with a PD-1/PD-L1 inhibitor combined with radiotherapy (*P* < 0.05), whereas the baseline NK cell count and IL-17A level were not significantly associated with PFS (*P* > 0.05). The details are presented in Table 2.

**Table 2:** Univariate and multivariate analysis of absolute count of lymphocyte subsets, cytokines and PFS in patients

Variable		Univariate analysis			Multivariate analysis		
		HR	95%CI	*P* value	HR	95%CI	*P* value
Baseline CD8 + T lymphocyte (count/uL)	< 249	0.257	0.095–0.693	0.007	0.328	0.130–0.826	0.018
	≥ 249						
Baseline T lymphocyte (count/uL)	< 730	0.797	0.325–1.951	0.619			
	≥ 730						
Baseline NK cell (count/uL)	< 359	0.382	0.149–0.976	0.044	0.399	0.154–1.033	0.058
	≥ 359						
Baseline IL-6 (pg/mL)	< 9.5	2.018	0.818–4.980	0.128			
	≥ 9.5						
Baseline IL-10 (pg/mL)	< 3.6	1.091	0.447–2.663	0.848			
	≥ 3.6						
Baseline IL-17A (pg/mL)	< 9.8	3.519	1.346–9.198	0.010	2.848	0.985–8.232	0.053
	≥ 9.8						
Baseline γ-INF (pg/mL)	< 1.8	1.896	0.782–4.595	0.157			
	≥ 1.8						

With respect to OS, univariate analysis revealed that lower baseline CD8 + T lymphocyte and NK cell counts were associated with worse survival outcomes (*P* = 0.011; *P* = 0.010), whereas higher baseline levels of IL-6 and IL-17A were associated with increased mortality risk (*P* = 0.003; *P* = 0.009). On the basis of these findings, baseline CD8 + T lymphocyte counts, NK cell counts, and the levels of IL-6 and IL-17A were selected for multivariate analysis. The results of the multivariate analysis revealed that the baseline CD8 + T lymphocyte count and IL-17A level were independent prognostic factors for OS in patients treated with a PD-1/PD-L1 inhibitor and radiotherapy (*P* < 0.05). In contrast, baseline NK cell counts and IL-6 levels were not significantly associated with OS according to the multivariate analysis (*P* > 0.05). The details are shown in Table 3.

**Table 3:** Univariate and multivariate analysis of absolute count of lymphocyte subsets, cytokines and OS in patients

Variable		Univariate analysis			Multivariate analysis		
		HR	95%CI	P value	HR	95%CI	P value
Baseline CD8 + T lymphocyte (count/uL)	< 249	0.348	0.154–0.787	0.011	0.374	0.151–0.929	0.034
	≥ 249						
Baseline T lymphocyte (count/uL)	< 730	0.808	0.367–1.781	0.598			
	≥ 730						
Baseline NK cell (count/uL)	< 359	0.339	0.149–0.774	0.010	0.414	0.161–1.064	0.067
	≥ 359						
Baseline IL-6 (pg/mL)	< 9.5	3.681	1.556–8.707	0.003	2.119	0.775–5.792	0.143
	≥ 9.5						
Baseline IL-10 (pg/mL)	< 3.6	1.452	0.659–3.200	0.355			
	≥ 3.6						
Baseline IL-17A (pg/mL)	< 9.8	3.620	1.380–9.496	0.009	2.873	1.054–7.829	0.039
	≥ 9.8						
Baseline Î^3^-INF (pg/mL)	< 1.8	1.682	0.743–3.810	0.212			
	≥ 1.8						

### Safety assessment

The incidence rates of adverse reactions in the PD-1/PD-L1 inhibitor combined with chemotherapy group, PD-1/PD-L1 inhibitor combined with tyrosine kinase inhibitor group, and PRaG therapy group were 77.1%, 58.3%, and 66.7%, respectively. The incidence rates of Grade 3 adverse reactions in the PD-1/PD-L1 inhibitor combined with chemotherapy group, PD-1/PD-L1 inhibitor combined with tyrosine kinase inhibitor group, and PRaG therapy group were 28.6%, 13.9%, and 16.7%, respectively. All adverse reactions reported in the safety analysis are presented in Supplementary Table 2.

## Discussion

The ORR, mPFS, and mOS for patients receiving combined therapy were 15.8%, 3.4 months, and 9.1 months, respectively. Compared with patients treated with immunotherapy alone, patients treated with combined therapy appeared to derive greater benefit. In this study, survival data analysis revealed that patients receiving PRaG therapy experienced superior survival outcomes compared with those in the other two treatment groups. No significant difference in OS was observed across the three groups, potentially due to limited sample size and insufficient follow-up time. Previous studies have demonstrated that ionizing radiation possesses immunomodulatory properties. Ionizing radiation can directly damage tumor cell DNA, leading to tumor cell death. Simultaneously, dying tumor cells release tumor-associated antigens (TAAs), inducing immunogenic cell death (ICD) and generating an in situ tumor vaccine effect [21]. Furthermore, ionizing radiation can promote the infiltration of immune cells into the tumor microenvironment, strengthening the recognition and cytotoxic activity of T lymphocytes and natural killer cells against tumor cells and thus increasing the systemic efficacy of immune checkpoint inhibitors (ICIs) [22, 23]. In this study, patients in the combined radiotherapy group received 10–24 Gy/2–3 f of large-fractionated radiotherapy. This approach, involving multisite radiotherapy for primary and metastatic lesions, not only facilitates full activation of the immune system but also minimizes the severe depletion of lymphocytes commonly observed with conventional fractionated doses and avoids the safety concerns associated with high-dose fractionation [24, 25]. Additionally, cytokines such as GM-CSF and IL-2 were incorporated into the treatment regimen. Previous studies have shown that GM-CSF promotes the polarization of monocytes into M1 macrophages and the activation of dendritic cells, both of which increase antigen presentation and amplify the immune response [26]. IL-2 also stimulates the antitumor activity of CD8 + T cells and NK cells [27]. The addition of these cytokines likely further augments the synergistic effects of immunotherapy and radiotherapy, which may explain why PRaG therapy yielded better therapeutic outcomes in this study.

Research on the molecular and biological mechanisms underlying PRaG therapy is ongoing. In this study, we further explored independent factors that may influence the therapeutic outcomes of PRaG therapy on the basis of clinical data. The antitumor efficacy of immunotherapy largely depends on the effective activation of CD8 + T cells and NK cells. Peripheral blood lymphocyte levels can serve as a rough indicator of immune function. Our study revealed that the baseline peripheral blood lymphocyte count was an independent prognostic factor for both PFS and OS in patients treated with PRaG therapy. Patients with higher baseline lymphocyte counts demonstrated better survival outcomes and improved treatment efficacy.

Absolute counts of peripheral blood lymphocyte subsets were measured before and during treatment in patients from the PRaG therapy group, providing an indirect picture of immune status during treatment. CD8 + T cells, which are critical effector cells in the antitumor immune response, constitute the cornerstone of immune-mediated tumor elimination [28]. In the subgroup analysis of the PRaG therapy group, the baseline CD8 + T cell count was identified as an independent prognostic factor for both PFS and OS. Patients with higher baseline CD8 + T cell counts exhibited more favorable survival outcomes.

Cytokines are a diverse class of proteins synthesized by both immune and nonimmune cells. In the context of antitumor immune responses, certain cytokines play an activating role in the immune system, whereas others suppress immune function. IL-6 is a well-known inflammatory cytokine that not only participates in various inflammatory responses but also plays a crucial role in inflammation-related cancers. IL-6 is a key regulator of myeloid-derived suppressor cell (MDSC) aggregation and activation and promotes tumor cell proliferation, survival, invasion, and metastasis. MDSCs, which accumulate during tumor progression, inhibit the antitumor activity of T cells and NK cells, thus contributing to tumor immune evasion. Consequently, IL-6 is associated with poor outcomes in cancer patients and is linked to adverse outcomes of immunotherapy [29]. IL-10, expressed by tumor-associated macrophages, is another cytokine involved in immune escape mechanisms in several malignant tumors. IL-10 contributes to the formation of an immunosuppressive tumor microenvironment (TME) in cancers such as gastric, ovarian, bladder, and pancreatic cancers and is correlated with poor patient outcomes [30–32]. IL-17A, a proinflammatory cytokine, induces the production of inflammatory mediators, upregulates PD-L1 expression, and reduces the efficacy of immunotherapy [33, 34].

In our study, a significant positive correlation was found between IL-6 and IL-10 levels in peripheral blood [35]. Blocking both IL-17A and IL-10 can increase the tumor response to PD-1/PD-L1 inhibitor immunotherapy in patients with MSS mCRC. Subgroup analysis of the combined radiotherapy treatment group revealed that elevated baseline IL-6 levels were associated with poorer survival outcomes, with patients with high baseline IL-6 levels having shorter survival times. Additionally, the baseline IL-17A level was identified as an independent prognostic factor for OS, with higher levels correlating with a poor prognosis. No significant association was found between baseline IL-10 levels and patient outcomes. It may be attributed to population differences and temporal factors.

The overall safety of this study was manageable. It is worth noting that in the PRaG group, two patients developed grade 3 immune checkpoint inhibitor-related pneumonia, and one patient experienced radiation pneumonitis. The KEYNOTE-001 study showed that patients who received radiotherapy had a higher incidence of pulmonary toxicity than those who did not (13% vs 1%, *P* = 0.046) [36]. Both pneumonitis patients in this study had lung metastases and received irradiation to lung lesions. Therefore, special attention should be paid to safety when combining radiotherapy for lung lesions with immunotherapy in PRaG treatment.

## Conclusion

PD-1/PD-L1 inhibitor-based therapies demonstrate efficacy in MSS/pMMR mCRC, with PRaG therapy showing advantages over chemotherapy/TKIs. In PRaG-treated patients, high CD8 + /low IL-17A correlated with superior outcomes.

## Supplementary Information

Below is the link to the electronic supplementary material.

Supplementary file1 (PDF 261 KB)

## Data Availability

The original contributions presented in the study are included in the article. Further inquiries can be directed to the corresponding authors.

## Abbreviations

mCRC: Metastatic colorectal cancer
MSS: Microsatellite-stable
pMMR: Proficient mismatch repair
PD-1: Programmed cell death protein 1
PD-L1: Programmed cell death ligand 1
GM-CSF: Granulocyte–macrophage colony-stimulating factor
PFS: Progression-free survival
OS: Median overall survival
NGS: Next-generation sequencing
ECOG: Eastern cooperative oncology group
RECIST: Response evaluation criteria in solid tumors
CTCAE: Common terminology criteria for adverse events
CR: Complete response
PR: Partial remission
SD: Stable disease
PD: Progressive disease
PET: Positron emission tomography
CT: Computed tomography
MRI: Magnetic resonance imaging
ECT: Emission computed tomography
ORR: Objective response rate
DCR: Disease control rate
TAAs: Tumor-associated antigens
ICD: Immunogenic cell death
ICIs: Immune checkpoint inhibitors
IL-2: Interleukin-2
IL-6: Interleukin-6
MDSC: Myeloid-derived suppressor cell
L-10: Interleukin-10
TME: Tumor microenvironment
IL-17A: Interleukin-17A
