# Supplementary material for: Efficacy of PRaG therapy in microsatellite-stable metastatic colorectal cancer: a comparative analysis of PD-1/PD-L1 inhibitor-based combination therapies
Source: Cancer Immunol Immunother. 2025 Oct 6;74(11):327. doi: 10.1007/s00262-025-04185-y (PMC12501090; doi:10.1007/s00262-025-04185-y)
Supplement: Supplementary file 1 — Supplementary file1 (PDF 261 KB) [file 262_2025_4185_MOESM1_ESM.pdf]

Supplementary Table 1 Evaluation of the efficacy of three groups patients with MSS/ pMMR mCRC

Group	ORR	DCR
Combined with TKI	16.7%	38.9%
Combined with chemotherapy	11.4%	28.6%
PRaG therapy	20.0%	50.0%

Supplementary Table 2 Adverse reactions in patients with MSS/ pMMR mCRC

Adverse events	All grade	Combined with chemotherapy (n=35)	Combined with TKI (n=36)	PRaG therapy (n=30)	Grade ≥ 3
Fatigue	49 (48.5%)	21	11	17	3 (3.0%)
Anorexia	38 (37.6%)	16	9	14	0
Leukopenia	27 (26.7%)	13	6	8	3 (3.0%)
nausea and vomiting	26 (25.7%)	13	7	6	0
Thyroid dysfunction	24 (23.8%)	8	7	9	0
Fever	20 (19.8%)	9	5	6	0
Liver dysfunction	17 (16.8%)	9	3	5	2 (2.0%)
Rash	15 (14.9%)	5	6	4	2 (2.0%)
Renal dysfunction	15 (14.9%)	6	3	6	2 (2.0%)
Anaemia	13 (12.9%)	6	2	5	1 (1.0%)
Thrombocytopenia	11 (10.9%)	6	2	3	3 (3.0%)
Diarrhea	9 (8.9%)	4	3	2	0
Reactive cutaneouscapillary endothelial proliferation	9 (8.9%)	3	4	2	0
hand-foot syndrome	9 (8.9%)	4	3	2	0
Pruritus	7 (6.9%)	3	2	2	0
Mouth Ulcers	5 (5.0%)	2	2	1	0
Immune Checkpoint Inhibitor-Related Pneumonitis	2 (2.0%)	0	0	2	2 (2.0%)
Radiation-induced pneumonitis	1 (1.0%)	0	0	1	1 (1.0%)
Autoimmune myocarditis	2 (2.0%)	0	0	1	1 (1.0%)

Grade 3 or higher adverse reactions in the combination chemotherapy group included Fatigue, Leukopenia, Thrombocytopenia and Liver dysfunction. Grade 3 or higher adverse reactions in the combination TKI group included Liver dysfunction, Renal dysfunction and Rash. Grade 3 or higher adverse reactions in the PRaG therapy group included Autoimmune myocarditis, Immune Checkpoint Inhibitor-Related Pneumonitis, Anaemia and Radiation-induced pneumonitis.
